# Opportunities in Open Science With AI

**DOI:** 10.3389/fdata.2019.00026

**Published:** 2019-09-27

**Authors:** Kuansan Wang

**Affiliations:** Microsoft Research, Redmond, WA, United States

**Keywords:** open science, big data, microsoft academic graph, artificial intelligence, research assessment

## Abstract

Bolstered by ever affordable computational power and open big datasets, artificial intelligence (AI) technologies are bringing revolutionary changes to our lives. This article examines the current trends and elaborates the future potentials of AI in its role for making science more open and accessible. Based on the experience derived from a research project called Microsoft Academic, the advocates have reasons to be optimistic about the future of open science as the advanced discovery, ranking, and distribution technologies enabled by AI are offering strong incentives for scientists, funders and research managers to make research articles, data and software freely available and accessible.

Throughout our history, scientists advance the state-of-the-art by building on the work from others. As famously put by Sir Isaac Newton, if we can see farther “it is by standing on the shoulders of giants.” The pivotal role of freely flowing knowledge in the progress of our science and technology naturally gives rise to the Open Science movement that aims at eliminating access barriers to scholarly communications (Open Access, OA), research data (Open Data), and the protocols and other software tools that gather and process the data (Open Source). Recent decades have seen scientists, research managers, and funding agencies embrace the Open Science idea with concrete actions. On OA, many have declared mandates and increased spending (Solomon and Björk, 2012; Pinfield and Middleton, 2016), contributing to the growing number of OA articles (Piwowar et al., 2018). Citations received by OA articles are markedly higher (Li et al., 2018; Piwowar et al., 2018), and websites or browser extensions that help researchers self-archive and find OA articles proliferate (Piwowar et al., 2018). Even the academic publishers, who initially feared OA as detrimental to their businesses, have apparently found opportunities to innovate and produce new OA products and services. Among the most notable examples is AAAS, the publisher of *Science* magazine, that has publicly shifted its stance and launched an OA journal *Science Advances* in 2015 to join the OA movement. Within the few short years, the new OA magazine has published more than 2,600 papers from 20 thousand authors that jointly have received more than 50 thousand citations and show no sign of impacting its non-OA counterpart. Similarly in the Open Data and Open Source arenas, guidelines, mandates, and commitments have been made (e.g., Gleeson et al., 2017), and scientists of various fields have started to utilize software development systems such as GitHub as a medium to exchange and archive necessary artifacts for their research (Perkel, 2016). An online journal (Rougier et al., 2017) has emerged to publish work dedicated to reproducing the discoveries reported previously elsewhere.

## Lingering Doubts About Open Science

Amidst these activities depicting an unmistakable trend, however, it is worth noting that the movement is still not universally welcome, as exemplified by the debates published as recently as in 2014 (Gibbs, 2013; Osborne, 2015). Among the issues raised against Open Science are worries that the movement can unleash into the public domain unprecedented amount of materials beyond our capacity to process them, thereby degrading the peer review quality and adding more stress on the discoverability and spread of new knowledge. Some are concerned that the data and the results can be misused and misinterpreted by unintended audience, especially when the data underlying some scientific claims are highly contextualized. Open Science is also tangled with the complex issues of how research is evaluated and funded. Many scientists surveyed by Mann et al. (2009), though identified with Open Science in principle, have not made any action plan to share their data and software tools because the existing research funding and evaluation structures offer no incentives to justify the extra efforts to circulate their resources.

## Understanding Scientific Outputs With AI: What Is Already Possible

Since 2014, however, the world has witnessed remarkable technological advancements, particularly in the field of artificial intelligence (AI) powered by the ever-easier access to big data and cloud computing. They enable us to take a scientific approach to study the structure, the evolutions and the societal impacts of science itself at a scale that is unimaginable only a decade ago (Fortunato et al., 2018). One enabling resource is from a research project at Microsoft Research. The project pushes the boundary of machine cognition technology by deploying software agents trained with natural language understanding capabilities to continuously scavenge the Web for research artifacts and, from them, extract up-to-date academic knowledge into a graph based representation called Microsoft Academic Graph (MAG) (Sinha et al., 2015). As of April 2019, the intelligent agents behind MAG have been versed in more than 660 thousand topics in more than 210 million academic publications and patent applications, spanning over two centuries and growing at an annual compound rate over 9%. As the records are from the entire web, MAG equalizes the discoverability of research materials made accessible by the incumbent publishers as well as by individual authors self-archiving at their own websites, potentially making policy initiatives to favor “Gold” over “Green” OA (e.g., Gibbs, 2013) less critical. Most notably, MAG is constantly watching the evolutions of fields of study to adjust its taxonomy and recategorize the publications it contains (Shen et al., 2018). Articles describing work in similar areas can be dynamically identified even though they are from authors of very different fields and do not cite one another or share any references (Kanakia et al., 2019). Such a feat, with a computational complexity of 210 million raised to the second power, would be prohibitively expensive with human labor but can now be economically achieved with modern computing infrastructure developed for big data and AI.

### Leveraging AI for Cognitive Overflow

**Figure 1:**
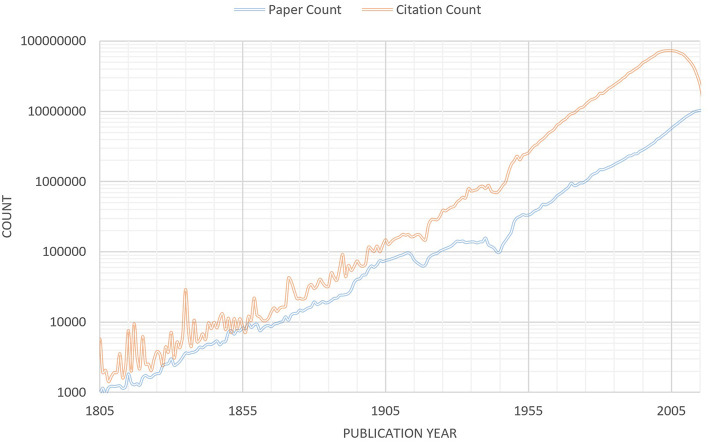
Number of publications (lower) and the citations received (upper) vs. the year of the publication based on the April 2019 snapshot of MAG. Since MAG sourced its data from the web, publications, and their citations not uploaded to the web may be under-represented. The widened gap between the two curves suggests publications in recent years have received more citations on the average. Note that publications newer than 2005 do not have sufficient time to gather their full due citations. The source code to reproduce this graph can be found at February 8, 2019 Microsoft Academic blog at https://www.microsoft.com/en-us/research/project/academic/articles/cost-of-tracking-research-trends-and-impacts-with-microsoft-academic-graph/.

With MAG being publicly available, researchers can take a closer look and examine various arguments on Open Science with data. Figure 1 (after Dong et al., 2017), for example, shows the research output in terms of number of articles published each year has been growing exponentially (with the exceptions of two world wars and major economic crisis in our societies) for almost two centuries, long before the modern Open Science movement was conceived. Furthermore, the growth is better explained by the increasing number of scholars (Dong et al., 2017) which, again, starts a century before the Open Science movement. It is therefore precarious to argue that Open Science has anything to do with it. The data do confirm the perception of information overflow: the world first sees the annual publications exceeding 1 million in 1974 and, today, that amount of new articles are being published in a month. Even zooming into individual fields of study, the evidence of information overflow is plentiful. If we use 10^4^ annual new publications, roughly 30 a day, as a human “cognition threshold” for individual scientists to keep up with the latest developments in their fields, there have been 383 such fields that have exceeded our “cognitive threshold” as of 2018. The two focal areas behind MAG, “Big Data” and “Deep Learning” (a subfield of AI underlying the natural language capabilities), have both entered this category in recent years (see Supplementary Material). Figure 1, in the meantime, also shows that papers published recently are being cited more, not less. Since papers need to be first found before they can be read and cited, this suggests their discoverability is not hindered by the large volume of publications in recent decades. Aside from better uses of technology, another possible explanation may be from the observation that the average number of authors per paper has also grown (Dong et al., 2017): papers with two or fewer authors account for 96% a century ago but <40% today, and scientists have been steadily expanding their collaboration circles, by 8-folds since 1950s. In other words, scientists are forming larger teams for a more complex research landscape, likely as a response to compensate individual cognitive overflow. Collaboration is a behavior unique to homo sapiens that, through the course of evolution, has given us decisive advantages over other species, including our close relatives in the same genus (Harari, 2011). Since successful collaborations are premised upon open communications, this evolutionary behavior of ours can be expected to further accelerate the movement toward Open Science and drive the opposite to extinction.

### Impacts Through Data and Software Sharing Are Already Recognized

Technologies are also changing how research contributions can be recognized in the era of Open Science (Piwowar, 2013). For example, text in the scientific literature contains attributions and commentaries that are a form of “crowd-sourced” peer reviews on the cited work. Frequently, the cited work refers not only to articles but also to datasets and software. Large scale text and data mining techniques, not available until recently, can now be applied to analyze and assess the importance of the cited work based on the collective judgments of the entire scientific community. For instance, the default ranking function in MAG is a network centrality measure developed by economists and social scientists that has been known to be powerful and contributed to Google's success in the form of its PageRank algorithm (Microsoft Academic FAQ). Using this ranking function, the most important work in Computer Science is the programming language for big data analytics R, and a software tool in machine learning called LIBSVM is ranked higher than the top article describing an algorithm of finding scale-invariant features in computer vision. In the field of protein structure, the highest ranked results are software tools such as SIR2004 and SWISS-MODEL, and the dataset Protein Data Bank is so impactful that it prompts the machine learning algorithm in MAG to recognize it as a subfield on its own. A recent survey (Candela et al., 2015) shows more research communities have recognized the value of software and data papers, and MAG data corroborate that publishers are responding with new journals dedicated to reports of these resources. This type of articles, when made widely discoverable, have an additional benefit to prevent and detect potential misuses of scientific data and tools because when the guidelines and terms of use are clearly prescribed they can be applied to more easily check against misuses. The answer to preempt and combat misuses of scientific artifacts, a concern on Open Science, is therefore to make science more open, i.e., to make how to use them even more transparent rather than obscure. As the impacts of making research resources available can be clearly identified with a simple metric, scientists who underestimate the payoff of making their data and software more widely available are misinformed, short-sighted and, more importantly, are missing the opportunity to be recognized as making larger and broader impacts than just their papers alone.

## What Is Likely to Come

Currently, the Open Science movement clearly benefits the publication industry and the scientists who want to access published materials. However, the cost of publishing is still not falling fast enough for many scientists. Highly regarded journals often charge as high as five thousand dollars for publishing an article, and even newer alternatives that cut the charge by an order of magnitude can still be out of reach to scientists from less affluent regions that have a lot to share with the rest of the world. This might be just a natural outcome of the economic law of supply and demand where highly sought-after publication venues can demand higher prices. There are many remedies to this problem, and we see the new discovery capability enabled by the technologies is one of them. Based on the citation behaviors, MAG has shown that scientists are increasingly treating preprint articles in the same way as those that have undergone peer review and been published in prestigious venues. It is no longer uncommon that an article with a future publication date in some traditional venue has already accumulated tens or even hundreds of citations because it is already widely read in its preprint form. In a way, a low cost yet effective medium for scientific discourse is emerging outside of the publication industry. Granted, there is no guarantee that these services will remain low cost, but the web search engine based solutions have the reach to the entire web to quickly identify wherever and whenever an alternative appears. Coupled with the improving natural language understanding techniques that enable high precision recommendations, the modern technologies have the potential to make high quality materials readily discoverable even though they are not present on the traditional distribution channels. Encouraged by the individual preprint articles being able to stand out on their scientific merits alone without the prestige of their eventual publication venues, we have reasons to be optimistic that the future Open Science will foster an even more inclusive and vibrant community where the new discoveries are streaming in from every corner of the world and technology advancements can be developed in a much more frictionless and economic fashion and applied to where they are most needed.

## Data Availability

Publicly available datasets were analyzed in this study. The dataset described in the article is free and open to the public but is distributed only through the Azure platform. This data can be found here: https://docs.microsoft.com/en-us/academic-services/graph.

## Author Contributions

The author confirms being the sole contributor of this work and has approved it for publication.

### Conflict of Interest Statement

KW was employed by the company Microsoft Research.
